# Contaminants in Grain—A Major Risk for Whole Grain Safety?

**DOI:** 10.3390/nu10091213

**Published:** 2018-09-02

**Authors:** Frank Thielecke, Anne P Nugent

**Affiliations:** 1 Swiss Distance University of Applied Sciences, Althardstrasse 60, Regendorf-Zürich CH-8105, Switzerland; 2 School of Biological Sciences, Queens University Belfast, 02.0014 Northern Ireland Technology Centre, Cloreen Park, Belfast BT9 5HN, Northern Ireland; A.Nugent@qub.ac.uk

**Keywords:** whole grain, contaminants, risk mitigation, mycotoxin, metal, human health, diet

## Abstract

Grains are the main energy and carbohydrate sources for human nutrition globally. Governmental and non-governmental authorities recommend whole grains as a healthy food choice. The role of contaminants in (whole) grains and how to mitigate any potential risk following their consumption has not been reported. With this narrative review, we shed light on the potential human health risk from contaminants in whole grains and elaborate strategies to mitigate such risk. We found that grains represent a significant source of food-borne contaminants, the main ones being; mycotoxins including (A) aflatoxin B1; (B) ochratoxin A; (C) fumonisin B1; (D) deoxynivalenol; (E) zearalenone; toxic metals like arsenic, cadmium and lead; as well as process contaminants such as acrylamide. Whole grains usually contain more contaminants than refined products. However, whole grains also provide more nutrients that may reduce the impact of these contaminants. Strict regulatory thresholds aim to minimize the risk of contaminants to public health. The consumer can further impact on the mitigation of any risk by eating a healthy diet filled with nutrient-dense foods such as whole grains and probiotics. The risk posed by contaminants from whole grains do not outweigh the known nutritional benefits of whole grain consumption.

## 1. Introduction

Globally, there are over 50,000 edible plants. Just three of these (rice, maize and wheat) provide about 60 per cent of the world's food energy intake [1]. Grains have a long history of use by humans, dating back to prehistoric times. Admittedly, pancakes and bread were still a long way off during the Middle Stone Age; however, evidence suggests that some humans in Africa at that time (i.e., 105,000 years ago) did indeed eat cereal-based snacks [2]. Nowadays, cereal grains are food staples and represent the primary source of carbohydrates worldwide. The Food and Agriculture Organisation (FAO) forecasts that world grain utilization in 2018/19 will reach a record level of 2646 million tonnes [3].

Along with being our main energy and carbohydrate sources, grains also naturally contain contaminants and, as a food category, represent one of the main dietary sources of foodborne contaminants. Various organisations have issued definitions of contaminants including The Codex Alimentarius Commission (CODEX) and the European Food Safety Agency (EFSA). The core of these definitions is “any substance not intentionally added to food which is present in such food as a result of the production, manufacture, processing, preparation, treatment, packing, packaging, transport or holding of such food or as a result of environmental contamination” [4]. The potential sources for the contamination of grains are mostly environmentally based and include air, dust, soil, water, insects, rodents, birds, animals, microbes, humans, storage and shipping containers, and handling and processing equipment. Most contamination is of a microbiological nature but heavy metals and process contaminants play a role, too. The secondary metabolites produced by fungi which can grow on grain (or mycotoxins) belong to the most toxic contaminants occurring in a wide range of food commodities [5].

Several definitions for whole grain have been suggested [6,7]. They agree largely on the statement “whole grains consist of the intact, ground, cracked or flaked caryopsis (kernel) after the removal of inedible parts such as the hull and husk. The principal anatomical components—the starchy endosperm, germ and bran—are present in the same relative proportions as they exist in the intact kernel”. The Healthgrain Forum definition acknowledges in addition that some parts of the grain, such as the outermost layers, are removed during processing to eliminate potential contamination of the outer bran [6]. Grains make a significant contribution to our macro- and micro-nutrient intake and high consumption of grains, particularly whole grains, have been associated with decreased risk of developing several chronic diseases. There is a growing body of evidence showing that people who consume more whole grains have a lower risk of some chronic diseases compared with people who include few whole grains in their diet [8,9,10]. Consequently, whole grains and foods made from them, provide greater carbohydrate quality, and are increasingly recommended as replacements for foods that are made from refined grains [11].

Furthermore, whole grains are also growing in popularity with consumers, with the term “whole grain” becoming synonymous with the words “wholesome” and “natural”. Consumer Insight data also suggest a surge in interest for “healthy” grains, including traditional and more “ancient” whole grains as part of a growing ‘healthy living’ trend [12], but perhaps also reflecting an uptake of dietary recommendations. Whilst there is some discussion regarding the potential for contamination of grains and grain products in the human diet and their safety of consumption as part of regular diets, typically this has tended to focus on a single contaminant or group of contaminants. Although infectious agents of disease (e.g., bacteria pathogens) are potential hazards in grain [13], they are not the major focus of this review which includes the likely most relevant contaminants for grains, namely mycotoxins, heavy metals and the process contaminant, acrylamide. In the nutritional sciences, the notion of a food structure or matrix and how that may confer health benefits has been described for commodities such as dairy products [14]; however, it is less well described for whole grain, particularly in terms of mitigating any potential risk from contaminants. Hence, with this paper we shed more light on the potential risks, if any, following dietary consumption of contaminants from whole grains. We will outline strategies currently in place to lessen any such risk and question whether they may be inherent properties within whole grains, which may mitigate or reduce risk as part of a healthy balanced diet. Although contaminants in animal feed may pose a risk for food, in this paper we focus on food for human consumption only.

## 2. The Role of Whole Grains for Health

Whole grains are nutrient dense and research demonstrates that the healthiest diets (those associated with reduced risk of non-communicable diseases such as cardiovascular disease) are characterised by a higher intakes of fruit, vegetables, nuts and legumes, and whole grains and lower intakes of red and processed meats [15]. Specifically for whole grains, evidence from epidemiological and human intervention studies suggests the promotion of whole grain foods over refined grain foods [16]. Observational studies provide consistent evidence of an inverse relationship between whole grains and cardiovascular disease [9] and type 2 diabetes [17]. There is also emerging evidence of a role for whole grain in reducing obesity [18] and cancer [19]. A recent prospective study found that whole grains reduced the risk of all-cause mortality and death [20] while several meta-analyses confirm a robust inverse association between whole grains and cardio-metabolic disease [8,10]. In contrast, human intervention studies yield mixed results and do not always support the strong and consistent epidemiological evidence. Such inconclusive findings from human intervention studies are possibly due to differences in study design such as duration of the study, population chosen (ill or healthy), type of whole grain chosen, and compliance with the test diet [21]. According to the Global Burden of Disease (GBD) study in 2016, a diet low in whole grains resulted in almost 4 million Disability Adjusted Life years (DALYs or the sum of years lost due to premature death and years lived with disability) in the European Union (EU) in 2016 [22]. The GBD study also estimated that almost 270,000 avoidable deaths from all causes in 2016 in the EU arose from a diet low in whole grains, of which 250,000 were deaths from cardiovascular disease. Overall, the balance of such evidence currently has led governmental authorities and scientific organisations to issue guidance and dietary recommendations encouraging whole grain consumption [11]. These recommendations in turn have resulted in practical guidance for consumers in the form of on-pack product health claims in the US [23] and labelling suggestions by expert organisations such as the Healthgrain Forum in Europe [24] and the Whole Grains Council in the US [25].

## 3. Types and Nature of Contaminants

**Figure 1:**
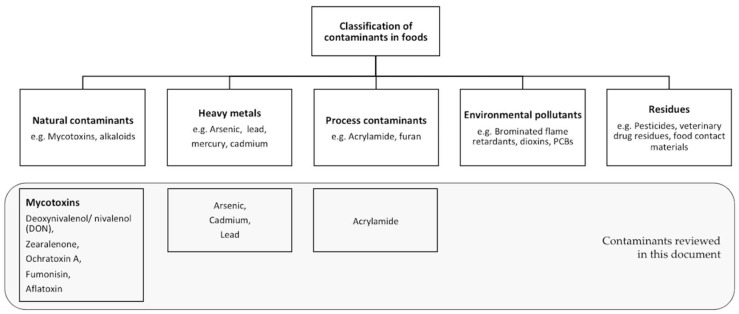
Classification of contaminants in food.

There is a myriad of contaminants naturally present in our foods that can be potentially harmful for human health. EFSA suggests following classification of contaminants in food (Figure 1) [26]. For the purpose of this review we focus on the, in grey, highlighted contaminants that are most relevant for grains, namely mycotoxins, heavy metals and the process contaminant, acrylamide. While we acknowledge residues (e.g., pesticides) may also be present as contaminants in cereals, a recent EFSA sampling report (2016) on pesticides demonstrates that 96% of samples analysed were within the limits permitted in EU legislation [27]. A global perspective on pesticides in grains may warrant a separate paper.

### 3.1. Mycotoxins

Mycotoxins are poisonous metabolites produced by certain species of fungi. Fungi are air borne or soil borne and can infect the plants already in the fields as well as throughout the production chain [28,29]. One of the earliest recorded example of poisoning caused by mycotoxins is ergotism, dating back as far as 600BC to records from Assyria [30]. Up until the 1950s, the infection of rye by ergot posed a significant public health issue in Europe and continues to do so today in less well developed parts of the globe, particularly in tropical regions. Ergot is a fungal disease of cereal grasses, especially rye, caused by species of the ascomycete fungus claviceps. There are about 50 species of claviceps, which can affect a wide range of grains including rye, wheat, sorghum, millet and barley, less often oat. Of note, ergot also became famous because it contains lysergic acid, a precursor for the synthesis of the illicit drug LSD. Worldwide, occurrence of mycotoxins remains a significant concern to human health [31,32].

**Figure 2:**
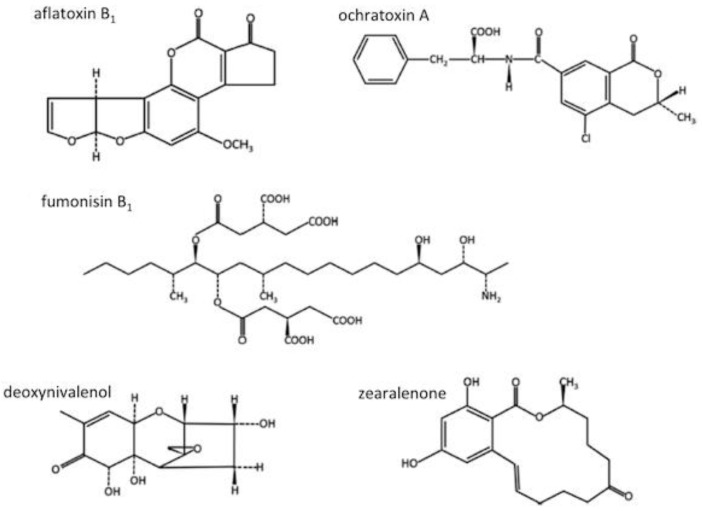
Mycotoxins in cereal grains, modified from [31].

In grains, five groups of mycotoxins are considered highly relevant for human health with their chemical structures depicted in Figure 2: deoxynivalenol/nivalenol (DON); zearalenone; ochratoxin; fumonisins; and aflatoxins. The concentrations of mycotoxins tend to be lower in processed food products; however, incidence rates can vary depending on the individual mycotoxin, possibly due to the varying stability of mycotoxins during processing and distribution. Lower levels of mycotoxins in food products are likely also due to manufacturers carefully selecting grains with low levels of mycotoxins. Global occurrence data reported during the past 10 years, reveals mycotoxin incidence rates among the positive samples identified and maximum levels in raw grains as 55% and 1642 μg/kg for aflatoxins, 29% and 1164 μg/kg for ochratoxin A, 61% and 71,121 μg/kg for fumonisins, 58% and 41,157 μg/kg, for deoxynivalenol, and 46% and 3049 μg/kg for zearalenone [31]. It is not reported if the incidence rates in the raw grains have changed during this 10 year period but current mycotoxin incidence rates clearly indicate that mycotoxins remain a concern, globally. Given that fungal growth on grains depends on various aspects, including temperature, pH, water availability, nutrients, and light, it is not unreasonable to suggest that climate may be a crucial factor and have a direct influence on the levels of mycotoxins present; however, no clear consensus exists. Using the example of DON contamination on wheat and corn, data from two different climate models failed to show clear increases in DON concentrations with the projected earlier maturation of the grains [33]. Other modelling attempts, from e.g., the EMTOX project (2012), disagree and point to a general increase of DON contamination in wheat in North-West Europe due to climate change [34]. In support, a modelling approach for aflatoxin B_1_ aiming to predict *aspergillus flavus* growth and aflatoxin production in corn in Europe concluded that aflatoxin B1 contamination in maize in Europe will increase due to climate change [35]. Given the differences in model parameters and huge variability in the results, a clear prediction of the influence of climate change is not possible, yet.

Table 1 summarises the staple grains and seeds that fungi affects, the fungal species that produce them, and the main effects observed in humans, with further details of each mycotoxin below.

#### 3.1.1. Deoxynivalenol/Nivalenol

Deoxynivalenol (sometimes called vomitoxin) is a type B trichothecene that occurs predominantly in grains such as wheat, barley, oats, rye and corn, and less often in rice, sorghum and triticale. Although being one of the less acute toxic mycotoxins, it is of particular interest due to its high prevalence and, as such, has been suggested as a potential marker of the occurrence of other mycotoxins [38]. This mycotoxin thrives in cool, wet conditions, with levels present in grains reduced but not eliminated by processing [39]. It is reported to withstand high temperatures ranging from 170 °C to 350 °C [38].

#### 3.1.2. Zearalenone

Zearalenone is heat stable and is found worldwide in a number of crops such as corn, barley, oats, wheat, rice and sorghum. Alternating low and moderate temperatures during storage favour zearalenone production, which has an optimum at 27 degrees Celsius. Zearalenone has been identified as a possible human carcinogen, possibly through its ability to act as an endocrine disruptor [40]

#### 3.1.3. Ochratoxin A

Ochratoxins in grains are found worldwide but can also occur in commodities like cereals, coffee and dried fruit. It is of special interest as it can be accumulated in the meat of animals, thereby finding its way into the human food chain through this additional route. Ochratoxin exposure in humans is linked with nephropathies [41].

#### 3.1.4. Fumonisin B1

Fumonisins were discovered as recently as 1988 and there is currently little information on their toxicology. Fumonisin contamination is commonly found in maize and maize products. High levels of fumonisins are associated with hot and dry weather, followed by periods of high humidity [42]. *F. moniliforme* growing in maize may produce fumonisin B1, a suspected human carcinogenic. 

#### 3.1.5. Aflatoxin

Aflatoxins (B1, B2, G1 and G2) are considered to be the group of mycotoxins of greatest concern from a global perspective [43], due to their wide distribution in a number of products and potent liver carcinogenic effect. Aflatoxins are classified as a Group 1 human carcinogen by IARC. Favourable conditions for growth of aflatoxins include high moisture content and high temperature. Aflatoxin B1 is the major toxin in cereal grains, such as corn, corn silage and sorghum. Aflatoxins are considered the most potent naturally occurring carcinogen. Since 2004, multiple aflatoxicosis outbreaks have been reported worldwide, resulting in 500 acute illness and 200 deaths. These outbreaks occurred mostly in Africa and were due to the consumption of contaminated home grown maize [44].

### 3.2. Metals as Contaminants

Heavy metals are found naturally in the earth. Arsenic, cadmium, lead, and mercury are toxic elements that are omnipresent at low concentrations in the environment [45]. They can enter plant, animal, and human tissues via inhalation, diet, and manual handling. Toxic heavy metals such as cadmium, mercury, lead and arsenic can not only compete with minerals such as calcium, magnesium or iron for absorption but can also bind to vital cellular components, such as structural proteins, enzymes, and nucleic acids, where they can interfere with their functioning [46,47].

Chronic exposure to heavy metals can lead to wide-ranging health problems. For example, arsenic affects the skin, lungs, brain, kidneys, liver, metabolic system, cardiovascular system, immune system, and endocrine system. Cadmium impacts the bones, kidneys, liver, lungs, testes, brain, immune system, and cardiovascular system [48].

#### 3.2.1. Cadmium

Cadmium is a toxic trace element found as an environmental contaminant, both through natural occurrence and from industrial and agricultural sources. Foods are the main source of cadmium exposure, second to smoking. Wheat and rice are major cadmium contributors to the diet. Cadmium is stored in the endosperm, the source of white flour. Grain genotypes vary and can influence the level of grain cadmium accumulation [45], with some genotypes exceeding the CODEX standard (0.2 mg/ kg). Especially in rural and economically-deprived populations in India [49], Africa [50], and China [51], cadmium is a concern for food security. 

#### 3.2.2. Arsenic

Arsenic is a ubiquitous element, which is introduced to the environment from both natural and anthropogenic sources. The toxicity of arsenic compounds strongly depends on their chemical forms—inorganic arsenic is considered to be more toxic than the organic form. All plants can absorb some arsenic, but rice, cultivated in flooded conditions, can absorb much more than other grains. On average, a 30-fold increase in arsenic has been reported for rice in flooded as opposite to nonflooded conditions [45]. The soil in the fields, when covered with water, creates conditions that allow arsenic to be converted to more readily absorbable forms, although the actual amounts eaten in the diet will depend on type of rice, growing, processing and cooking conditions [52].

Arsenic accumulates most in the outer layer of rice, which is the reason that whole grain rice, with its bran intact, can have up to 80% more arsenic than white rice [53]. Assessments by EFSA have concluded that rice is safe to eat by all population groups as part of food-based dietary guidelines [54,55]. It is also safe to eat by infants and young children, who have higher intakes of arsenic relative to their body size than older children and adults. In Europe, maximum safety limits have only been applied to rice and rice-based products with a monitoring programme for arsenic occurrence in a wide variety of foods, including cereal grains, due for completion in 2018 [56]. The Swedish Food Standards Agency has advised that rice cakes should not be consumed by children under 6 years. However, neither EFSA nor any other European countries have considered this currently a necessary approach [57].

#### 3.2.3. Lead

Lead is also an ubiquitous element, found naturally in the earth’s crust at an average level of 10 mg/kg. In addition, lead is used in various industrial applications and can thereby be introduced for example, to flour by old mills or cracked grindstone with metallic lead [58,59].

There does not appear to be large differences in the location of lead stored in different compartments of the grain. In a case report from Albania, lead in flour was 325 ± 18 ppm, while in the bran it was 370 ± 22 ppm. The level in flour was sufficient to result in ~0.42 ± 0.05 ppm in the blood of the exposed individuals [59]. There is no known safe blood lead level, but chronic exposure to lead of the above levels can seriously harm particularly a child’s health.

#### 3.2.4. Mercury

The levels of mercury in the earth’s crust are usually around 0.02 mg/kg. Like arsenic, this element occurs in organic and inorganic forms. The organic form is considered most toxic. Mercury is not of major concern for grains but rather for seafood, hence we will not further discuss mercury here.

### 3.3. Acrylamide as Process Contaminant

Acrylamide is present in the diet of most people. Its formation relies on the presence of carbohydrates (reducing sugars such as glucose and fructose), the amino acid asparagine and heat. Therefore, fried and baked products like coffee, potatoes, bread and breakfast cereals can be a significant source of acrylamide. Overall, it is estimated that the average consumption of acrylamides, depending on population and age, is between 0.3–2.0 microg/kg body weight [60]. Levels of acrylamide in products range substantially and depend not only on processing but also on growing conditions of the crops and methods used for analysis. 

The IARC have classified acrylamide as potentially carcinogenic [36], hence the EU Commission updated the benchmark values for acrylamide levels in various food categories in 2017 [61]. In addition, the acrylamide levels are to be as low as reasonably achievable (ALARA principle). The US FDA proposes acrylamide levels in food but does not regulate them [62].

## 4. Magnitude of Mycotoxin Contribution from (Whole) Grains 

Taking the FAO number of approximately 25% of food crops being affected by fungi, the loss of foodstuff by mycotoxins is estimated to range around 1 billion tonnes per year and cause economic losses of billions of dollars worldwide each year [63]. Grains, alongside meats, milks and fruit and vegetables represent key agricultural trading commodities, but they also play key roles influencing diet and health as reflected by their frequency and extent of consumption captured well in dietary survey data. Using data from the UK population dietary intake survey, the National Diet and Nutrition Survey (NDNS), the relative (%) contribution of grain and grain products to energy and nutrient intake can be viewed as considerably greater than that for meat, milk or fruit and vegetables (Table 2) [64].

In the NDNS, for energy alone, cereal and cereal products contributed approximately 2% to 3.5% more to population energy intakes than meats, milks or fruit and vegetables, with similar values for the other nutrients listed. Extracting the absolute contribution of whole grain cereals and whole grain containing foods is unfortunately more difficult to quantify as the whole grain content of foods is often recorded from food ingredient listings rather than from food composition tables as with other nutrients [24], and intakes are not routinely published as part of many national food consumption surveys, such as with the NDNS. Given the potential presence of contaminants within these grain foods, their relative contribution to the intake of food contaminants is also regularly assessed to ensure public health safety. Unsurprisingly, as grain-based foods can contain low levels of contaminants but are consumed in significant quantities by large sections of the population, they can have a greater impact on contaminant exposure assessments, as reflected in recent EFSA opinions for metals such as cadmium [65] or arsenic [55]. Nevertheless, absolute concentrations of such contaminants are typically as low as possible and often lower than comparable concentrations for other commodities e.g., nuts, fruits, vegetables or starchy tubers. Ultimately, this means that a risk to public health from whole grain products is relatively low and ensures their continued promotion as part of food-based dietary guidelines e.g., “basing meals on potatoes, bread, rice, pasta or other starchy carbohydrates, ideally wholegrain” as recommended by the UK Eatwell plate [66].

Although this paper focuses on grains for human consumption, the possibility also exists for carry-over of the contaminants present in grains fed to animals as part of ‘rations’ or ‘concentrates’ to foods of animal origin destined for human consumption. Hence, food of animal origin can also represent a potential risk and can contribute to mycotoxin intake in humans. For example, it is long known that aflatoxin B1 in dairy cattle feed can be metabolised to aflatoxin M1 in milk [67]. Such co-contamination is discussed in greater detail elsewhere [68].

## 5. Strategies to Mitigate the Risk of Contaminants for Human Health

**Figure 3:**
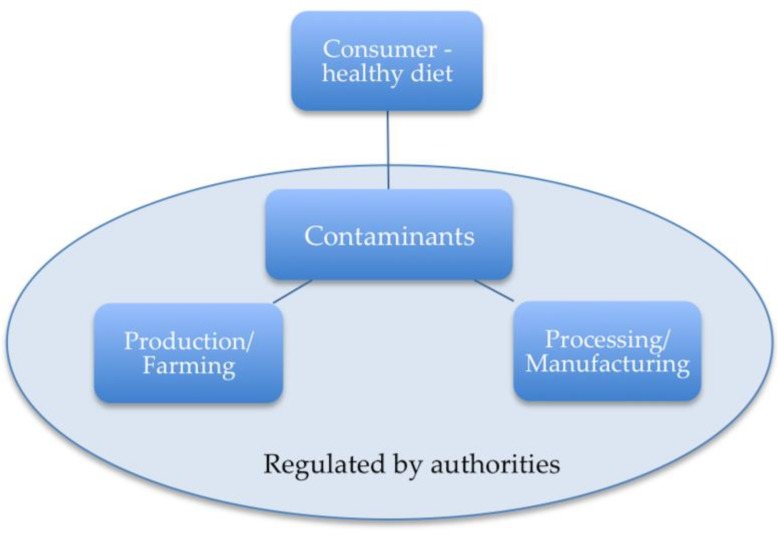
Important players to mitigate the impact of contaminants.

There are obvious sectors along the food production chain that can impact the risk of contaminants for human health, including production/farming, processing, distribution, retail and out-of-home production. Within these sectors there are a myriad of steps, which are not covered in detail here but warrant a separate publication. For the purpose of this publication, we confine ourselves to the sectors illustrated in Figure 3. An important role in this simplified approach falls to the appropriate authorities, who provide a regulatory framework for managing any risk. We have identified here that production/farming and processing/manufacturing are two other crucial sectors that influence the risk of contamination.

Fundamentally important, however, is the consumer and the fact that an overall healthy diet can play a significant role in mitigating the risk of contaminants in grain.

### 5.1. Regulations

Global and regional or national organisations such as Codex Alimentarius, the European Food Safety Authority, national food and drug administrations [69] (e.g., US FDA) have outlined principles for ensuring that maximum levels of contaminants in food or feed are as low as reasonably possible through Good Agricultural Practice and Good Manufacturing Practice [4,70]. Further, in addition to stating actions to prevent or reduce food contamination, risk assessment and risk management strategies are outlined should they be needed following identification of a health hazard after consumption of a contaminated food [70]. Detailed ‘Codes of Practice’ for the prevention and reduction of contamination such as mycotoxins in cereals [71] or lead in foods [72] have also been developed. As many contaminants are naturally occurring, it would be impossible to impose a total ban on their presence. Hence, most competent organisations and countries responded to the threat of food-borne contaminants by establishing and enforcing maximum levels for mycotoxins in food that are technologically practicable [73]. Within Europe, such levels are set on the basis of scientific advice provided by the European Food Safety Authority [74]. However, individual Member States are responsible for sampling foods to ensure compliance. For imported foodstuffs, the country of origin is responsible for compliance with EU legislation and this is checked at EU borders and on the market [4]. Other comparable competent authorities include the US Food and Drug Administration, and with the Canadian Food Inspection Agency in Canada. Table 3 provides a summary overview of the regulatory limits and guidance values issued by the EU and US competent authorities for mycotoxins, metals and acrylamide.

There are differences in the approaches and regulatory limits applied between the two jurisdictions with the EU authorities appearing to follow a more conservative approach. Exact comparisons are difficult due to differences in food grouping categories used; however, it seems that overall the numerical maximal limits applied to contaminants in grain and grain products for human consumption are somewhat lower in the EU than in the US for mycotoxins such as aflatoxin, fumonisin and deoxynivalenol. Further, while limits were established for heavy metals such as cadmium and lead in the EU, no such limits have been developed as yet in the US. The two jurisdictions also differ with their approach to acrylamide with benchmark values developed for the EU, but no values set as yet for the US. Such differing approaches may present challenges to farming communities and global food business operators; however, it is important to emphasise that rigorous monitoring is completed by both US and EU authorities and enforcement is enacted where required to ensure the highest food safety standards across the food chain and to use the best scientific advice to protect public health.

### 5.2. Farming/Production

Cultivation of the crops is the first step in managing the level of contaminants. Temperature and moisture content of the grain or commodity are the most critical factors favouring fungal growth and mycotoxin production. In general, moulds grow at a temperature range of 10 °C to 40 °C, above 70% relative humidity and a pH range of 4 to 8. Relative humidity is another factor influencing the moisture content of stored grain resulting in more or less water available for mould growth and subsequent mycotoxin production. Hence, growing conditions and storage of the grains are the main preventive measures to avoid fungal growth. This includes avoiding factors that cause crop stress such as insect damage, bird damage, drought stress, and early harvest, as well as avoiding kernel damage during harvesting, transporting. Mycotoxin content also increases with delayed harvest coupled with rain and cool periods. Other farming practices can influence risk e.g., maize and wheat are particularly susceptible to *Fusarium* and should not be rotated close to each other. Codex practice guides recommend appropriate crop rotation to lessen risk e.g., by including potatoes, other vegetables, clover, alfalfa in rotational cycles [71].

With respect to heavy metals, increases in understanding of the molecular pathways responsible for the accumulation of arsenic and cadmium have been made, leading to the development of crop varieties with significantly reduced concentrations of toxic metals in their edible parts [45]. Other agronomic strategies to reduce the toxic metal accumulation in grains include liming; the application of organic material to reduce the bioavailability of metals such as cadmium in the soil; silicon fertilization to saturate the transport pathway that mediates arsenic accumulation; and the optimal management of irrigation regimes to control cadmium and arsenic bioavailability [82].

In the formation of acrylamide in cereal products, asparagine is the critical component. Research has shown that free asparagine content varies widely between grain varieties and even within one single grain variety. Further, it is also influenced by the growing (soil) conditions in individual fields. For example, sulphur-deprived soils have been shown to impact the free asparagine concentrations in certain cereal crops considerably. Less sulphur in the soil results in higher asparagine levels in the crop and therefore higher risk of acrylamide formation [83]. Overall, because of the heterogeneous nature of this variation (i.e., grain type, variety, growing conditions, climate), it is almost impossible to source wheat or any other grain with controlled low levels of asparagine.

#### Organic vs. Conventional Farming

Given that synthetic fungicides and mineral fertilisers are not used in organic production, the question arises as to whether organically prone crops may be more vulnerable to fungal infection and mycotoxin contamination than conventionally grown crops [84,85,86]. A number of studies have been completed in this regard with a recent review of the available data from controlled field trials, farm surveys and food basket surveys of cereal crops reporting no significant difference with respect to deoxynivalenol [87], zearalenone, ochratoxin and fumonisin contamination between the two farming systems [88]. Lower levels of Ht-1+T-2 toxins (two of the most toxic members of the trichothecene group, the same group as DON) were observed in organic than conventionally grown oats. The same is true for zearalenone, where organically grown corn showed lower levels [89]. However, the same study reported higher levels of aflatoxin in organically grown corn as compared with conventionally grown corn. Cirillo et al. (2003) evaluated conventional and organic food products based on corn, wheat and rice and reported the median levels of mycotoxins to be similar between organic and conventional products [90]. Generally, it was noted that levels of mycotoxins present were low in both systems, often below analytical levels of detection and well below the EU limits for food. Weather conditions, years, locations, tillage practice and crop rotation were regularly reported as more influential than farming type. Overall, the authors concluded that neither of the farming systems increased the risk of mycotoxin contamination [88]. 

### 5.3. Processing/Manufacturing

Once the grains are harvested, a prime role in the mitigation of the risk of contaminants falls to the processing/manufacturing steps. Whole grains undergo thorough cleaning and dehulling, with debranning (as is done for refined grain) applied to a limited extent in order to retain the nutritional superiority.

Large fractions of mycotoxins can be removed by sorting, cleaning, dehulling, and debranning reduction of damaged kernels, fine material, and dust [91]. Appropriate cleaning of the harvested grain is essential to reduce mycotoxin content, as concentrations can be greatest in broken kernels and fine material. Furthermore, proper drying has to follow soon after harvest. Grain dried below 14% moisture content can arrest further mould growth and mycotoxin production. However, it will not eliminate fungi and mycotoxins that are already present. The following moisture contents are considered safe during storage: 14% to 14.5% for wheat, barley and oats; 14% for corn; and 13% to 14% for rice. Another strategy is to prevent fungi growth storing grains in an atmosphere with high carbon dioxide concentrations >80% [92]. In contrast, mitigation of acrylamide formation is mainly achieved through changes in product composition and/or process conditions, which in turn may have an impact on the nutritional quality (e.g., decreased nutrient bioavailability, changed flavour, taste/palatability, texture). Fermentation is a key processing step in bread making; extended fermentation time has been shown to result in bread dough with lower levels of acrylamide [93]. Other process-related parameters are temperature and oven time. For example, in non-fermented crisp bread, reduction in process temperature and oven speed reduced acrylamide by approx. 75% [93]. In contrast, one composition approach is to replace ammonium bicarbonate with sodium bicarbonate, which helps control acrylamide formation, but if applied systematically, will increase sodium levels. High sodium intake is a potential risk factor for cardiovascular disease [94]. A benefit-risk assessment should therefore be made for certain products on a case-by-case basis.

A second composition approach relates to asparagine. While the sugar compositions of grains are an important component in the formation of acrylamide, they seem to be of less impact than the asparagine level in wheat bread [95].

Choice of wheat with lower free asparagine has led to products with lower acrlyamide levels. However, current experience suggests that specifying low free asparagine wholegrain is not yet possible [83], but that using less whole meal (lower bran and germ) and more endosperm will be effective because asparagine is more concentrated in the germ/bran. However, this will significantly compromise the product’s organoleptic and nutritional properties.

Such processing can also affect the nutritional value of cereals in a positive way. For example, when grains are milled into flour, the intact botanical structures are opened, which lowers the amount of resistant starch (a type of starch that is not digested in the small intestine, therefore functions at a dietary fibre) [96]. Thus, milling makes the nutrients more available for digestion. During wet processing such as soaking, fermentation of dough or in the preparation of porridge, another component called phytate may be degraded (broken down). Phytate binds minerals like copper and iron (decreasing their bio-availability), so its degradation may increase the availability of such micronutrients for absorption and use in the body [97].

Current strategies to influence heavy metal composition typically relate to milling and cooking. For example, the milling of durum wheat reduced the presence of the elements nickel > arsenic > cadmium > lead [98]. There may also be potential positive effects of heat. During thermal processes such as baking and extrusion, phenolic compounds, which have antioxidant properties, are generated [99].

#### Treatment

If fungi and mycotoxins are present in grains, several methods have been developed to treat mycotoxin-contaminated grain, including ammonification and the use of gases such as chlorine dioxide, and sulphur dioxide. Ammonification of contaminated corn is one potential treatment option that has been widely used. It was found effective in reducing the fumonisin B1 levels in cultured and naturally contaminated corn by 30% and about 45%, respectively. The use of chlorine dioxide or sulphur dioxide gas at higher concentrations (500 or 1000 ppm) with longer exposure time (24 hours) has been found effective to a degree (as a structural fumigant) in arresting certain species of fungi. Alternative methods are the use of ozone (O_3_) and gamma-irradiation. However, there are several limitations associated with each method, and complete elimination of contaminants from food product by processing can rarely be achieved. Therefore, priority should always be given to prevention, which is more efficient than treatment [100].

### 5.4. The Role of Whole Grains as Part of A Healthy Diet for the Mitigation of Mycotoxins and Toxic Metals

With regulations in place that set standards for maximum levels of contaminants as a result of farming practices and the processing of grains, the question remains what can the consumer do in order to mitigate any potential risk from ingestion of contaminants from whole grains. Common hygiene standards as well as guidance for the consumer by the means of “date marks” are useful tools. For example, advising the consumer to adhere to the date mark, ‘use by’, may reduce likelihood of consuming any mould (a certain types of fungus) that may grow on bread that has been stored inappropriately: In this section, we outline dietary strategies and highlight the inherent properties within whole grains, which when consumed as part of a healthy balanced diet, may reduce the risk of contaminants.

Toxic metals compete with other metals for the transport systems in the plant, which explains why they are taken up into the different compartments of the grain. For example, cadmium is stored in the endosperm of wheat, the source of refined flour and to a lesser extent in the outer layers of the grain [101]. Fungi often colonizes the outer layers of the grains but some can also infect, for example, corn kernels through slit channels.

#### 5.4.1. Nutritional Content of Whole Grain vs. Refined Grain 

Compositionally, whole grains are more nutrient dense than refined grains [102]. It is the bran, the outer layer of the grain that contains fibre, antioxidants, B vitamins, phytochemicals, and 50–80 per cent of minerals in grains like iron, copper, zinc, and magnesium. The germ contains healthy fats, B vitamins, phytochemicals, and antioxidants like vitamin E. The endosperm, the largest layer contains mostly carbohydrates, protein, and small amounts of some B vitamins and minerals. Refining leaves only the endosperm, hence 50–80% of the phytonutrients and minerals get lost during this step. Table 4 shows the difference in nutritional value of whole grain versus refined grain [103]. Although poorly described, there is some evidence to suggest that the presence of these nutrients could potentially prevent some of the negative effects of contaminants in whole grain foods and will be described in more detail below.

#### 5.4.2. Mycotoxins

Dietary strategies suggested to contain the toxic effects of mycotoxins include the presence of antioxidant compounds (selenium, vitamins, provitamins), and other food components (phenolic compounds, coumarin, chlorophyll and its derivatives, fructose, aspartame) [104]. Generally speaking, foods with high levels of antioxidants, vitamins and carotenoids as well as superoxide anion scavengers, such as ferulic acid in whole grain wheat [105], have the potential to reduce the impact of mycotoxins by protecting cell membranes from mycotoxin-induced damage [106,107]. These nutrients and bioactives are not only found in whole grains but also in fruits and vegetables, which underlines a protective role for whole grains but in the context of an overall healthy, balanced diet.

The potential of probiotic microorganisms as a strategy to degrade mycotoxins has been shown in milk [108]. This effect by the probiotic microorganisms is associated with fermentation, antibiosis and the ability of the microbial cell wall to bind to the mycotoxin. Probiotic strains from kefir have been shown to bind mycotoxins and to decrease their gastrointestinal absorption. Specifically, the kefir grains adsorbed 82 to 100% of aflatoxin B1, zearalenone, and ochratoxin A, when cultivated in milk [109]. Although not currently widely consumed, kefir could be added as an ingredient to commonly consumed foods. Although preliminary in nature, such promising findings warrant further research into the potential of probiotics in the adsorption of mycotoxins. Further support for this approach is generated from studies which reveal the ability of microbes to efficiently bind aflatoxin B1 in grains such as corn, sorghum and rice [110].

#### 5.4.3. Toxic Metals

In humans, the first line of defence against toxic metals is at the site of absorption. For toxic metals and essential minerals to be absorbed, they must bind with transporters in the small intestines. Empirical data suggest optimum levels of nutrients reduce toxicity [111], which in turn may imply that if the consumption of minerals is optimal it might mitigate the toxicity of heavy metals. This hypothesis is supported by a body of animal and in vitro research which suggests a beneficial role for selenium, magnesium, calcium, zinc, and iron, and dietary ingredients e.g., dietary fibre in reducing uptake and toxicity of heavy metal such as cadmium and lead [111,112,113,114]. In a mouse model, it was shown that supplemental magnesium (40 mg/kg body weight) led to 30% percent reduction in cadmium retention in the liver compared with animals that were intoxicated with cadmium only [112]. Future research in humans should advance the understanding of the role of these bioactives in a grain matrix.

Dietary fibre has been suggested as a protective ingredient with wheat bran shown to bind heavy metals and other toxicants like heterocyclic amines [115]. Indeed, in a mouse model, wheat bran bound ca. 90% of the cadmium provided in the diet [116]. Additional in vitro studies show that dietary fibres prevent cadmium from entering mouse intestinal tissue [116], suggesting that dietary fibres have the potential to protect the body from toxic metals and should be explored further.

Some mechanistic studies are available to support a protective effect, showing interactions between essential minerals and heavy metals, particularly cadmium. In an animal feeding study, when rats were supplemented with magnesium (a mineral naturally found in whole grains, at a rate of 70 mg/kg body weight over 28 days) while being exposed intragastrically to cadmium chloride, a dose response effect of magnesium on the retention of cadmium was observed [117]. Specifically, the authors reported that magnesium stimulated the de novo production of the anti-oxidant glutathione, which would partly explain the protective effect of magnesium. Additional mechanistic studies are available showing an interaction between zinc and cadmium through the family of proteins called metallothionein. (Metallothionein is a family of metal-binding proteins that are important for a number of functions including zinc and copper homeostasis and buffering against toxic metals) [118]. Specifically, zinc increases the synthesis of metallothionein and hence, the ability to bind and excrete heavy metals. Further, toxic metals, especially cadmium, compete with zinc (and copper) for binding with metallothionein and displace it, stimulating further metallothionein synthesis due to the levels of free zinc. Another suggested mechanism for the protective effects of the essential minerals iron, calcium, magnesium, and zinc is that they can impede absorption of dietary cadmium, by down-regulating intestinal expression of mineral transporters, and by directly competing with cadmium for access to these transporters [119].

Collectively, these studies provide some indication of how dietary mineral intakes may mitigate the toxic effects of heavy metals. Nevertheless, most of the available data is mainly limited to cellular and animal studies and using elemental forms rather than whole foods, with few studies directly examining the effect of dietary deficiency or supplementation on metal toxicity [120]. Unsurprisingly, due to ethical reasons, human trials with contaminants are rare, so no strong evidence from randomized controlled trials is available, or likely to become available in future.

However, there is some epidemiological evidence which strongly suggests that iron deficiency is associated with increases in lead absorption [121,122]. Results from a cohort of 60 Egyptian children also reveal decreased iron absorption when blood lead levels were ≥10 μg/dL [123]. 

Further, an inverse relationship was observed between iron and arsenic levels as analysed in hair samples from 168 Taiwanese patients with blackfoot disease (blackfoot is a form of peripheral vascular disease common in parts of Taiwan) [124]. Here, the authors also suggested that iron has an antagonistic relationship with arsenic, which in turn suggests that sufficient iron levels mitigate the toxicity of arsenic. In the US, an analysis of NHANES data in 1–11 year children reported an inverse association between calcium deficiency and blood lead levels [125]. Overall, although the above data mostly rely on epidemiological and preclinical evidence, there is, therefore, some evidence to suggest the presence of many essential minerals, such as those present in foods such as whole grain, may help mitigate the risk from heavy metal contaminants. At the very least, there is no evidence to suggest that they increase any risk. Indeed, it has been suggested elsewhere that people eating a diet deficient in micronutrients will be predisposed to toxicity from nonessential (heavy) metals [120].

## 6. Final Considerations

This paper has primarily discussed the types of contaminants that may be present in whole grains and whole grain foods, the potential risks associated with their presence and the considerable number of strategies that are in place to lessen the likelihood of any harm to human health. We have also highlighted the properties inherent in whole grains which may mitigate risk, when consumed as part of a healthy, balanced diet. Other considerations, we believe, which merit mention include the fundamental role we as consumers must play in mitigating any risk from grains and ongoing research which seeks to find ways to reduce their presence and/or improve techniques used as part of risk analysis.

Fundamental roles that we as the consumer can take in mitigating the risk of contaminants from grains, include the simple practical step of avoiding foods that carry mould or are known to be high in toxic metals, where possible; such steps can of course be more difficult in areas with food poverty and/or food insecurity. We also acknowledge that, generally speaking, while processed foods tend to contain lower levels of contaminants, this is not always the case and there are instances that show higher levels in refined products, e.g., cadmium in refined flour. Further, processed foods can also be higher in less desirable ingredients from a public health nutrition perspective such as sugar, fat and/or salt. Focusing on rice, there has been some interest in the ability of a rinsing step by consumers to impact metal levels in rice. Unfortunately, rinsing rice had little impact; however, cooking rice in excess water (6–10 parts water to 1 part rice) and draining excess water is reported to reduce arsenic levels somewhat [126]. Rice grain type appears to impact somewhat, with a 40% decrease in arsenic content observed following cooking of long grain polished rice in excess water, while reductions of 60% and 50% were reported for parboiled and brown rice [127]. Of note, iron, folate, niacin and thiamin contents were also reduced by 50–70% for the polished and parboiled varieties, but significantly less with the whole grain brown rice [127]. Hence, high consumers of rice may wish to consider rice varieties and cooking methods in any attempt to reduce arsenic concentrations in rice [52].

Mention also needs to be given to the advances in monitoring and scientific methods underway that are not limited to reducing the presence of contaminants and/or to capitalise on new methods for assessing risk. Examples of European research projects include MYCHIF, funded by EFSA, focusing on mycotoxin mixtures in food and feed [128] or MyToolBox, funded by the European Union, which applies a field-to-fork approach to reduce moulds and mycotoxins in the food and feed chains in Europe and China (https://www.mytoolbox.eu). It is recognised that continued, and perhaps even greater, efforts will be needed by multiple stakeholders, particularly with the advent of global warming and climate change. Although not acceptable to European consumers, the ability of genetic engineering to reduce the presence of such contaminants in crops is recognised and utilised elsewhere globally [129]. There is also a need for more research in this area, not least to understand the potentially protective mechanisms by which essential minerals may lessen risk from heavy metals and how this is impacted by the food matrix within whole grains but also how this may interact with the other antioxidant compounds present in foods such as fruit and vegetables and to confirm whether probiotic microorganisms are able to bind mycotoxins when consumed as part of a healthy diet. Authors should discuss the results and how they can be interpreted in perspective of previous studies and of the working hypotheses. The findings and their implications should be discussed in the broadest context possible. Future research directions may also be highlighted.

## 7. Conclusions

Grains feed the world. However, along with being our main energy and carbohydrate source, grains are also a noteworthy source of dietary contaminants. The present review has explored the potential risk to human health of contaminants commonly found in grains, namely mycotoxins, toxic metals and acrylamide, and has commented on mitigation strategies in place. The complete elimination of such contaminants from grains or any other food product by processing can rarely, if ever, be achieved; hence prevention is more efficient than treatment. The value of guidance documents and safety standards as established by Food Safety Authorities around the world cannot be overstated in terms of protecting public health and providing guidance and assistance to stakeholders, not least farmers and processors. Further, as reassurance to the consumer, advances in many scientific disciplines mean that these food safety standards have never been as high as today and are adhered to right across the food chain. Refining grains will reduce the presence of many contaminants but it also removes 50 to 80% of phytonutrients from whole grains. The observational and preclinical evidence presented here suggests that it is these phytonutrients, (vitamins, minerals and fibres) that may exert a potentially protective effect against mycotoxins and toxic metals in particular. Further, the consumer also has a choice in mitigating any risk from contaminants, and to do so best by continuing to eat a healthy balanced diet, rich in nutrient dense foods, and including whole grain foods. Such a diet will ensure adequate trace mineral status and sufficient intakes of nutrients, antioxidant compounds, phenolics and dietary fibres. Current dietary recommendations for whole grain consumption can help the consumer to make these healthier choices.

## Figures and Tables

**Table 1:** Mycotoxins in staple grains and seeds.

Mycotoxin	Fungal source(s)	Effects of ingestion for humans	Commodity
Deoxynivalenol/nivalenol	*Fusarium graminearum*	Human toxicoses e.g. nausea, vomiting, diarrhoea, headache, fever	Wheat, maize, barley
	*Fusarium crookwellense*		
	*Fusarium culmorum*		
Zearalenone	*F. graminearum*	Identified by the International Agency for Research on Cancer (IARC) [36] as a possible human carcinogen.	Maize, wheat
	*F. culmorum*		
	*F. crookwellense*		
Ochratoxin A	*Aspergillus ochraceus*	Suspected by IARC as human carcinogen.	Barley, wheat, and many other commodities
	*Penicillium verrucosum*		
Fumonisin B1	*Fusarium moniliforme* plus several less common species	Suspected by IARC as human carcinogen.	Maize
Aflatoxin B1, B2	*Aspergillus flavus*	Identified as potent human carcinogens by IARC.	Maize, peanuts, and many other commodities
Aflatoxin B1, B2, G1, G2	*Aspergillus parasiticus*		Maize, peanuts

Modified from [37].

**Table 2:** Percent contribution to energy, macronutrient and fibre intake of UK adults aged 19–64 years. Data from NDNS–2014/2015 and 2015/2016.

Food Group	Energy	Protein	Fat	Carbohydrate	Fibre
All Cereal and cereal products	32	23	21	46	38
Meat and meat products	17	37	24	6	12
Milk and milk products	9	13	12	5	1
Fruit, vegetables & salad vegetables	9	7	6	11	28

**Table 3:** Comparison of regulatory guidance in the EU and US for foods for human consumption concerning (i) maximum levels of total mycotoxins permitted; (ii) maximum levels (EU) or guidance values (US) for metals; and (iii) benchmark levels (EU only) for acrylamide.

Contaminant	EU		US	
	Food Category	Maximum Level ** (ppb)	Food Category	Maximum Level ** (ppb)
**Mycotoxins**				
Aflatoxin	All cereals (inc. maize and rice) for direct human consumption	4	All foods except milk	20
	Baby foods and processed cereal based foods for infants and young children	0.1 for aflatoxin B1		
Deoxynivalenol	Cereal flour, maize flour, maize, grits and maize meal, dry pasta	750	Finished wheat products for human consumption	1000
	Bread, biscuits, pastries, cereal snacks and breakfast cereals	500		
	Processed cereal based baby and infant food	200		
Fumonisin	Maize and maize based foods intended for direct human consumption	1000	Degermed dry milled corn products (e.g. corn meal or corn flour with fat content < 2.25%, dry weight basis)	2000
	Maize based breakfast cereals and maize based snacks	800	Cleaned corn intended for popcorn	3000
	Processed maize based foods and baby foods for infants and young children	200	Whole or partially degermed dry milled corn products dry milled corn bran; cleaned corn intended for mass production	4000
Ochratoxin A	Cereal products and cereal grains intended for direct human consumption	3	None identified	
	Baby foods and processed cereal based foods for infants & young children	0.5		
Zearalenone	Cereals for direct human consumption (e.g., cereal flour, bran)	75	None identified	
	Maize for direct human consumption, maize based snacks & breakfast cereals	100		
	Bread, pastries, biscuits, cereal snacks, breakfast cereals	50		
	Processed cereal & maize based foods and baby foods for infants & young children	20		
**Metals**				
Cadmium	Cereal grains excluding wheat & rice	100	None identified	
	Wheat and rice grains, wheat bran & wheat germ for direct consumption	200		
Arsenic	Parboiled rice and husked rice	250	Infant rice cereals ***	100
	Rice waffles, rice wafers, rice crackers and rice cakes	300		
	Rice destined for production of foods for infants and young children	100		
Lead	Cereals (pulses & legumes)	200	None identified	
**Process Contaminant**				
Acrylamide ****	Soft bread–wheat	50	None identified	
	Non-wheat based soft bread	100		
	Breakfast cereals, excluding porridge:			
	(i) Bran and whole grain cereal, gun-puffed grain, wheat and rye based products	300		
	(ii) Maize, oats, spelt, rye barley and rice-based products	150		
	Processed cereal foods for infants and young children	40		

References [39,42,61,73,75,76,77,78,79,80,81]; ** maximum levels listed for total mycotoxin subtype unless stated e.g., aflatoxin values reflect those from both B1 and M1 subtypes unless stated; *** relate to action levels only with other considerations required prior to any enforcement by US authorities; **** EU Regulation values for acrylamide relate to benchmark levels only, they are not maximum limits.

**Table 4:** Nutritional compositions of different whole grain and refined grains, per 100 g *.

Nutrient	Whole wheat flour	White, wheat flour, 75% extraction	Rye flour	Rye flour, 60% extraction	Brown rice (raw)	White rice (raw)	Barley (whole grain raw)	Pearl barley
Carbohydrates, g (% of energy)	62 (75.6)	71 (80.6)	55 (71.4)	73 (85)	73.5 (82.4)	78 (87)	60.8 (72.8)	67 (79)
Protein, g (% of energy)	10 (12.2)	12.6 (14.3)	10 (13)	8 (9.3)	8.3 (9.3)	7 (8)	10.6 (12.7)	9 (10.6)
Fat, g (% of energy)	2 (5.5)	1.1 (2.8)	2 (5.8)	1 (2.6)	2.6 (6.6)	1 (2.6)	2.1 (5.7)	2 (5.3)
Dietary fibre, g	11	4	15	5	3	1.3	14.8	8.6
Vitamin B1 (Thiamine), µg	0.4	0.07	0.4	0.15	0.34	0.04	0.31	0.03
Vitamin B2 (Riboflavin), µg	0.35	0.04	0.2	0.07	0.03	0.03	0.10	0.03
Vitamin B3 (Niacin), mg	5.7	1	1.7	1	6.1	1	5.2	3
Vitamin B6 (pyridoxine), µg	0.35	0.12	0.22	0.23	0.25	0.12	0.56	0.25
Vitamin B9 (Folate), µg	37	22	78	28	49	20	50	20
Iron, mg	4	0.8	4	1.5	1.3	0.4	6.0	2
Zinc, mg	2.9	0.64	3	1.3	0.8	1.8	3.3	2
Magnesium, mg	124	20	20	92	51	157	13	44
Sodium (salt), mg	5	2	5	10	1	2	4	5

Modified from [103]. * Varies between products and countries.
